# Effect of Different Polymerization Degrees and Fatty Acids of Polyglycerol Esters on the Physical Properties and Whippability of Recombined Dairy Cream

**DOI:** 10.3390/foods12010022

**Published:** 2022-12-21

**Authors:** Guosen Yan, Shiran Wang, Yang Li, Jing Zhang, Hao Ding, Yan Li, Liebing Zhang

**Affiliations:** 1 Beijing Engineering and Technology Research Centre of Food Additives, School of Food and Health, Beijing Technology and Business University, Beijing 100048, China; 2 College of Food Science and Nutritional Engineering, China Agricultural University, Beijing 100083, China; 3 College of Engineering, China Agricultural University, Beijing 100083, China

**Keywords:** dairy product, recombined dairy cream, polyglycerol esters, whippability

## Abstract

Polyglycerol esters (PGEs) are used as emulsifiers in recombined dairy cream (RDC) to improve product quality. In this study, the effects of four PGEs with different polymerization degrees and esterification on the particle size, viscosity, zeta potential, and microrheology of RDC emulsions were investigated, and the whipping time, overrun, serum loss, and firmness of the RDC emulsions were recorded. The results show that the addition of the PGEs reduced the particle size (from 2.75 μm to 1.48–1.73 μm) and increased the viscosity (from 41.92 cP to 73.50–100 cP) and stability (from 0.354 to 0.105–0.128), which were related to the change in interfacial properties and the weakening of Brownian motion, but there were differences in the effect on the whipping behavior of the RDCs. Although the addition of 0.9% triglyceride monolaurate gave the emulsion the best stability, the RDC had a longer whipping time (318 s) and a lower overrun (116.6%). Comparatively, the 0.7–0.9% concentrations of PGE55 and tripolycerol monostearate (TMS) provided RDC with good stability and aeration characteristics, allowing inflation within 100 s and expansion rates of up to 218.24% and 186.88%, respectively. In addition, the higher degree of polymerization of polyglyceryl-10 monstearate (PMS) did not work well at any concentration. These results contribute to understanding the mechanism of action of PGEs and improving the quality of RDC.

## 1. Introduction

Whipped cream is a traditional dairy product that is widely used in desserts, pastries and ice creams, and it is very popular among consumers. Natural whipped cream is usually formed by dispersing 30–40% of milk fat in a serum aqueous phase consisting of water (58–74%), some milk proteins (2–3%), lactose, and minerals, and it is a typical oil-in-water system [1,2]. During the whipping process, the dispersed fat droplets encapsulate the churned air, thus forming a stable foam structure [3]. However, the aggregation and floating of fat droplets in natural cream via gravity lead to the loss of churning characteristics and a short shelf-life, presenting significant challenges during storage and transportation, especially for areas far from the milk source. Currently, manufacturers are using anhydrous milk fat, milk protein concentrates (MPCs) and other ingredients in standardized formulations of recombined dairy cream (RDC) unaffected by lactation season, region, transportation, etc. [4]. However, the destruction and reconfiguration of natural fat globule membranes during industrial production also pose new challenges in terms of product stability.

The amphiphilic nature of an emulsifier allows it to adsorb at the oil/water interface in order to compensate for the loss of the natural fat globule film and the increase in the specific surface area caused by industrial production and homogenization processes [5], reducing the interfacial tension and improving the stability of the fat globules in the aqueous phase [6]. In addition, there is competitive adsorption between the added emulsifier and the interfacial protein, which affects the interfacial properties of the mixed adsorption layer and plays a crucial role in emulsification and foaming stability [7].

Polyglycerol fatty acid esters (PGEs) are nonionic emulsifiers with excellent properties, consisting of a glycerol skeleton with different polymerization degrees and fatty acids, which determines their hydrophilic and lipophilic properties [8], and there are differences in the stabilization and emulsification of different types of PGEs in food. Uchino et al. developed PGE-based nanoparticles to deliver tocopheryl acetate and found that the addition of tetrameric glycerol laurate stabilized the nanoparticles and increased tocopherol accumulation in the epidermis [9]. Yoon et al. developed nanomicelles composed of fatty acids and monoglycerides and found that the nanostructure of monolaurate glycerides caused changes in membrane morphology [10]. It was also shown that the addition of long-chain saturated fatty acid emulsifiers, such as monoacylglycerol, increased the crystallization temperature and induced nonhomogeneous interfacial nucleation [6].

In general, the diverse molecular structures and types of PGEs make them versatile in terms of emulsification and stabilization, and they have great application potential in the modern food industry. However, the effects and mechanisms of PGEs with different fatty acids and polymerization levels on the quality of RDC have not been reported. In this work, we investigated the effects of different types of PGEs on the stability of emulsions and the churning process, assessed the performance and application prospects of different PGEs in RDC, and provided a basis for the selection of suitable PGEs for products with different characteristics.

## 2. Materials and Methods

### 2.1. Materials

**Figure A1:**
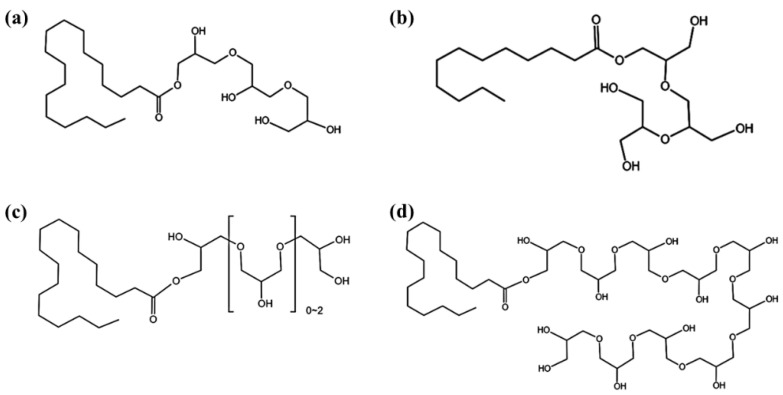
Molecular structure of different polyglycerol esters: (**a**) triglyceride monostearate (TMS), C27H54O8, molecular weight 506.71, hydrophilic and lipophilic balance 6.2; (**b**) triglyceride monolaurate (TML), C21H41O8, molecular weight 422.56, hydrophilic and lipophilic balance 8.5; (**c**) PGE55, C24H48O6–C30H60O10, molecular weight 432–580, hydrophilic and lipophilic balance 7; (**d**) polyglyceryl-10 monostearate (PMS), C48H95O22, molecular weight 1024.25, hydrophilic and lipophilic balance 11.3.

Anhydrous milk fat (Anchor, Auckland, New Zealand) and MPC powder (70.0% protein, Saishang Dairy Ltd., Ningxia, China) were commercially available. Triglyceride monostearate (TMS), triglyceride monolaurate (TML), and polyglyceryl-10 monostearate (PMS) were obtained from Wuhan Yuancheng Gongchuang Technology Co. (Wuhan, China), and PGE55 was provided by Danisco; their molecular structures are shown in Figure A1. Cellulose microcrystalline was obtained from Beijing Yinhexue Biotechnology Development Co., Ltd. (Beijing, China). All other reagents were chemically pure.

### 2.2. Formulation of RDC

A total of 36 g of anhydrous milk fat previously melted in a water bath at 70 °C was used as the oil phase; 2.86 g of MPC and 0.3 g of cellulose microcrystals dissolved in 50 mL of water were used as the aqueous phase. Furthermore, RDC stabilized by TML, TMS, PMS, and PGE55 was prepared by adding different proportions (0, 0.3, 0.6, 0.9, or 1.2 g) of emulsifier to the oil or aqueous phase. The two phases were mixed and fixed with water to 100 g, stirred at 500 r/min for 30 min (RW20, IKA, Stauffen, Germany) and subjected to a homogenization treatment at 4 + 2 MPa using a two-stage homogenizer (APV-200, Hötzelsdorf, Denmark), and then they were stored at 4 °C [11]. The preparation of RDC was repeated at least three times.

### 2.3. Average Droplet Size

The average size of the fat globules was determined using a laser diffraction-type particle sizer (SALD 3000, Shimadzu, Kyoto, Japan). Emulsions were diluted in deionized water at a ratio of 1:100, and particle size was measured at a shade of 40–60% and a refractive index of 1.460 at 25 °C [12].

### 2.4. Viscosity

The viscosities of the RDCs were determined by using a rotating concentric double-drum viscometer (DV-III, Brookfield, WI, USA). A total of 8 mL of the emulsion was added to the bucket and tested for 4 min at 30 r/min (39.6 s^−1^) and 25 °C using a SC4-18 rotor, and the values were recorded.

### 2.5. Physical Stability Analyzed Using LUMiSizer

The stability of the RDC was analyzed by using a LUMiSizer full-featured stability analyzer (LUM, Berlin, Germany), which accelerates the onset of instabilities, such as the aggregation, flocculation, and sedimentation of the emulsions, via centrifugation. The instability index was obtained by recording the change in the transmittance of NIR light at different positions in the cuvette with time [13]. The emulsion was slowly injected into the centrifuge tube (PC-110-131XX) to avoid bubbles, and the liquid level was flush with the standard scale. The test parameters were as follows: a temperature of 25 °C, a speed of 4000 r/min, a time interval of 10 s, and a measurement time of 120 min; 720 measurements taken.

### 2.6. Zeta Potential

The zeta potential of the RDC was determined by using a Malvern Zetasizer Nano90 (Malvern Instruments, Malvern, UK), following the method devised by Wei et al. with minor modifications [14]. All samples were diluted 500 times with deionized water, and they were added slowly to the cuvette (DTS1070) in order to avoid air bubbles.

### 2.7. Whipping Process and Overrun

A total of 200 g of RDC was placed in a churning bowl stored at 4 °C for 24 h, stirred at 1100 r/min for 30 s and then stirred at 1900 r/min (AHM-P125A, North American Electric ACA, Inc. Hernando, MS, USA) for rapid aeration until the cream could stand, and the churning time was recorded. The mass per unit volume of the RDC (m_1_) and the whipped cream (m_2_) was recorded, and the overrun was calculated as follows:

Overrun (%) = (m_1_ − m_2_)/m_2_ × 100. (1)

### 2.8. Serum Loss

Syneresis was used as an important indicator of foam stability: the lower the rate of cream dehydration and drainage, the higher the cream stability. The serum loss was determined by taking a certain mass of whipped cream (m_1_), placing it in a beaker with a sieve for 2 h at 25 °C, and recording the mass of the cream in the beaker (m_2_); the serum loss was determined using the following equation:

Serum loss (%) = m_2_/m_1_ × 100. (2)

### 2.9. Firmness

The texture of the whipped cream was determined using a texture analyzer (CT3, Brookfield, WI, USA) equipped with a TA3/100 probe, which was pressed down 20 mm at 10 mm/s with a trigger force of 2.5 g and then returned to its original position at the same speed [15].

### 2.10. Serum Protein Concentration

The amount of serum protein reflects the protein adsorbed by the fat globules to determine the condition of the interface. The determination of the serum protein was based on a slight modification of the method used by Zhou et al. [16]; 10 g of RDC was centrifuged for 60 min at 10,000 r/min at 4 °C, the serum and bottom protein were removed in a digestion tube and measured using an automatic Kjeldahl nitrogen tester (Kjeltec 8400, FOSS, Hilleroed, Denmark), and the serum protein concentration (T) was calculated as follows:

T (%) = P/M × 100,(3)

where P is the amount of protein in the serum and sediment (g), and M is the mass of the RDC (g).

### 2.11. Microrheological Properties

A Rheolaser Master microrheometer (Formulaction, Toulouse, France) was used to analyze the microrheological properties of the emulsions. In this study, 20 mL of RDC was poured into a cuvette, and the measurement started after the temperature of the test chamber reached 25 °C. The measurement time was 2 h [17]. In the measurement process, a laser with a wavelength of 650 nm was introduced into the microrheometer, and a multipixel detector collected the scattering map generated by the scattering wave to obtain the curve of the droplet mean square displacement (MSD) with time. The elasticity index (EI) and macroscopic viscosity index (MVI) were obtained using the software RheoSoft Master.

### 2.12. Fat Agglomeration Rate

A total of 0.005 g of Oleoresin O was added to 500 g of corn oil and slowly stirred for 24 h at 25 °C, protected from light to dissolve the Oleoresin O pigment, and stored under dark conditions [18]. A total of 10 g of the RDC and 10 g of the prepared Oleoresin O solution were mixed together and centrifuged at 10,000 r/min for 30 min. The upper layer of oil was then immediately transferred to a cuvette, and the absorbance was measured at 520 nm. The change in absorbance shows the mass of the unemulsified part of the fat:

(4) $$D=\frac{m_{0}\times (A-1)}{m_{1}\times \omega }$$

where *D* is the fat aggregation rate, *m*_0_ is the mass of the Oleomargarine O pigment (g), A is the ratio of the absorbance of the Oleomargarine O solution to the supernatant after centrifugation, *m*_1_ is the mass of the RDC (g), and ω is the fat content of the RDC (%).

### 2.13. Statistical Analyses

All experiments were performed at least three times to avoid errors, and the results are expressed as the mean ± SD. A one-way analysis of variance (one-way ANOVA) was performed using SPSS 20.0 (Chicago, IL, USA), and significant differences were considered when *p* < 0.05.

## 3. Results and Discussion

### 3.1. Effect of PGEs on Particle Size of RDC

**Figure 1:**
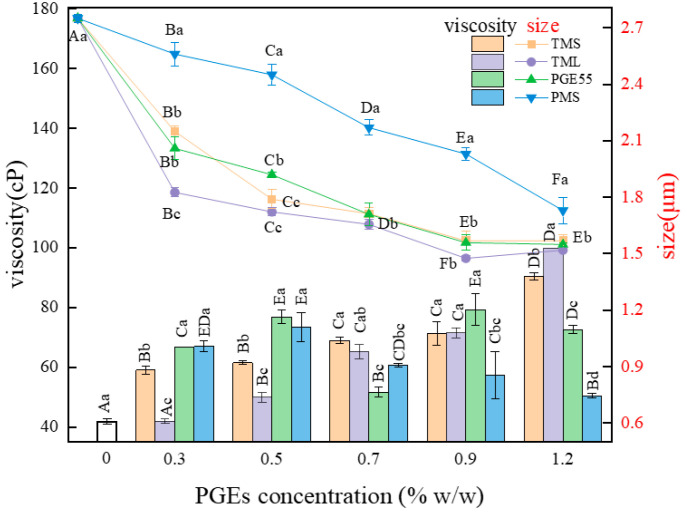
Effect of different PGEs on particle size (the line) and viscosity (the bar) of RDC emulsions. Uppercase letters indicate differences between concentrations, and lowercase letters indicate differences between PGEs.

The homogenization of RDC is a process in which the oil phase is uniformly dispersed in the aqueous phase as fat droplets, and the emulsifier is adsorbed on the surface of the fat droplets to maintain stability. The effects of the different PGEs and their concentrations on fat globule sizes in the RDC are shown in Figure 1. Generally, the addition of the PGEs significantly reduced the fat globule size, and the particle size decreased further with an increase in the concentration of all types of PGEs. This indicates that the PGEs with surface activity served as an emulsifier [6]. However, at high levels (0.9–1.2%), there was no statistical difference between the fat globule sizes (*p* > 0.05). In the absence of the PGEs, the fat globules were stabilized by surface-adsorbed interfacial proteins, but the proteins were less efficiently emulsified and formed larger droplets [19]. In contrast, PGEs can be tightly adsorbed at the oil/water interface, reducing interfacial tension and preventing the aggregation of fat globules; hence, a significant reduction in globule size occurs [20].

The effect of the different PGEs on the particle size of the emulsions varied, where the particle size of the emulsions with PMS (1.73–2.56 μm) was significantly higher than that of the emulsions with TMS (1.57–2.15 μm) at any concentration. This may be due to the higher polymerization of the main chain and the higher molecular weight of PMS, which was less effective in adsorption at the interface [21]. There were also differences between TMS and TML with the same degree of polymerization; the fat globule size of the TML emulsions at 0.3% was significantly lower than that of the TMS emulsions (*p* < 0.05), which may be related to the greater hydrophilicity of fatty acids with shorter carbon chains [22]. In addition, the fat globule size increased slightly (1.52 μm) at 1.2% TML concentration in the emulsion, probably due to the high concentration of emulsifier spilling over the oil/water interface and causing crosslinked aggregation between the fats [23].

### 3.2. Effect of PGEs on Viscosity of RDC

Viscosity reflects the spatial resistance to particle movement in the system and is closely related to the stability and inflation characteristics of the emulsion. The effects of the different PGEs on the viscosity of the emulsion are shown in Figure 1. The addition of the PGEs increased the viscosity of the emulsion in the concentration range of 0–1.2%, which was consistent with the addition of the PGEs reducing the fat globule size. The reduction in size increased interparticle interactions and spatial dislocation resistance, with a consequent increase in viscosity [24]. The concentration–viscosity relationships of the RDC emulsions prepared with the different PGEs varied. At low concentrations, there was no difference between PGE55 and PMS (*p* > 0.05), while PGE55 gradually increased the viscosity of the emulsion with an increase in its concentration, and, at concentrations >0.9%, the viscosity of the emulsion with PGE55 was significantly higher than that of the emulsion with PMS (*p* < 0.05). This suggests that the difference in the polymerization process affects the effect of the PGEs, which is consistent with the results of particle size. In contrast, the viscosity of the TML emulsion was significantly lower in the range of 0.3–0.7% (*p* < 0.05) than that of the other samples, although the degree of polymerization was the same as that of the TMS emulsion, which may be due to the shorter lauric acid fatty chain and stronger affinity with the aqueous phase [23]. Moreover, the viscosity of the TML emulsions at high concentrations increased rapidly and reached a maximum, presumably related to stronger interactions between the TML bound to the surface of the fat globule.

### 3.3. Effect of PGEs on Emulsion Stability of RDC

**Figure 2:**
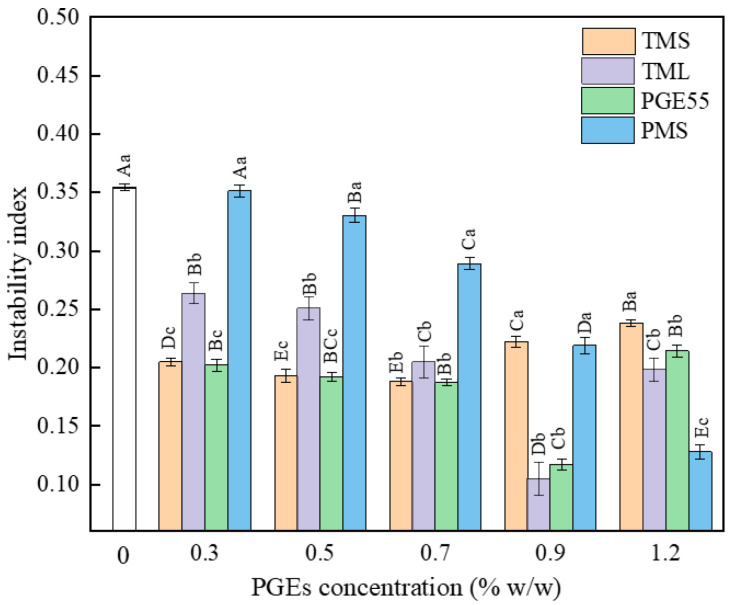
Effect of different PGEs on the instability index of RDC emulsions. Uppercase letters indicate differences between concentrations, and lowercase letters indicate differences between PGEs.

The stability of the emulsions prepared with the different PGEs was determined by using a multi-sample analytical centrifuge based on STEP technology. The instability index was determined using the integral transmittance at the sample position as a function of the centrifugal force action time, with larger values indicating more unstable emulsions [25]. The instability indices of the RDCs prepared with the different types of PGEs are shown in Figure 2. The TML and PGE55 emulsions had the lowest instability indices at 0.9%, compared to 0.7% for the TMS emulsion and 1.2% for the PMS emulsion. This indicates that the different types of PGEs had different optimum additions. In the range of 0–0.7%, the PMS with the highest degree of polymerization had the worst stability, while the PGE55 with the lowest degree of polymerization had the best stability; at 1.2%, the opposite result was observed. TMS and TML had different variations as a function of addition, with TML being more unstable at low concentrations and having the best stability at 0.9%, consistent with the trend in viscosity, indicating that differences in fatty acid type affect the performance of the PGE, which is consistent with the findings of Peng et al. [22]. Moreover, it has been previously shown that the movement of fat globules in emulsions can be explained by Stokes’ law [26] and that the instability index is positively correlated with fat globule size. In addition, the viscosity of the emulsion had a negative effect on the instability index. The small size and spatial site resistance reduced creaming, flocculation, and the coalescence of the fat globules due to Brownian motion [27]. The particle size and viscosity led to differences in the instability index of the PGEs, with the 0.9% TML emulsion having the best stability, consistent with the smaller particle size and higher viscosity.

### 3.4. Effect of PGEs on Zeta Potential of RDC

**Figure 3:**
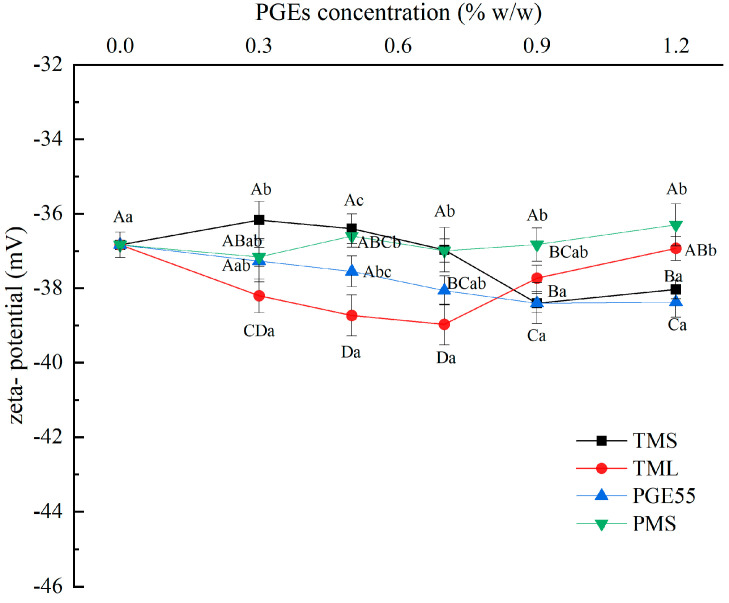
Effect of different PGEs on zeta potential of RDC emulsions. Uppercase letters indicate differences between concentrations, and lowercase letters indicate differences between PGEs.

The zeta potential indicates the magnitude of the particle charge and reflects the strength of the electrostatic interaction between particles; a stronger electrostatic repulsion contributes to the stability of particles [28]. The zeta potential values for the different samples are shown in Figure 3. In the absence of an emulsifier, the potential of the emulsion was negative (−36.83 mV) due to the adsorption of negatively charged milk proteins on the surface of the fat globule. The addition of the PGEs changed the adsorption state of the proteins at the interface, and this change differed among the different types of PGEs. At low concentrations, the potential of the TML cream was lower than that of the control, with the lowest potential at 0.7% (−38.97 mV), which may be due to a synergistic effect between TML and the protein, contributing to the adsorption of the protein at the interface and increasing the amount of interfacial protein [22]. In contrast, the addition of TMS at low concentrations (<0.7%) increased the potential, which was attributed to the gradual replacement of the interfacial proteins by TMS with better lipophilicity through the orogenic displacement theory, reducing the protein concentration; this indicates that the different fatty acids affected the interfacial adsorption behavior of the PGE [29]. However, at high concentrations (>0.7%), TMS and PGE55 showed the same trend of decreasing potential values, which may have caused stronger hydrophobic interactions between the emulsifier and the protein at the interface due to the lower degree of polymerization and the greater lipophilicity of TMS and PGE55, leading to further dissociation of the protein and the formation of more hydroxyl groups [30,31,32]. In addition, the effect of the addition of PMS on the potential was not significant, which may have been due to its weak interfacial adsorption capacity.

### 3.5. Effect of PGEs on Whipping Time of RDC

**Figure 4:**
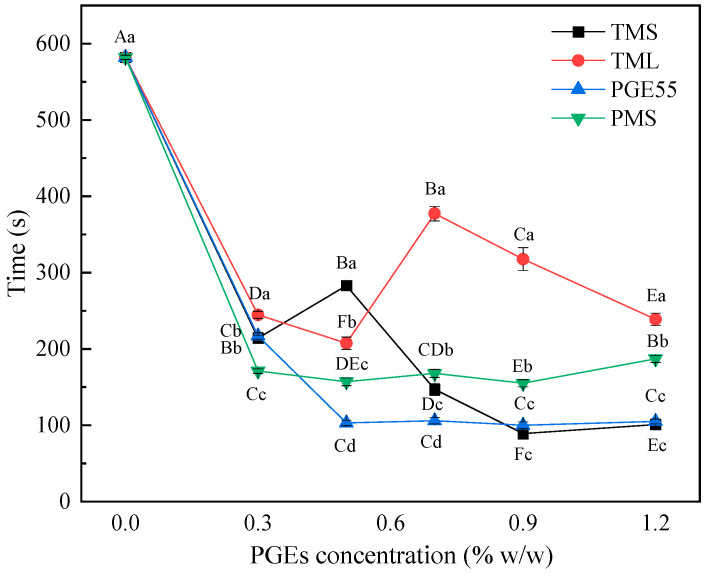
Effect of different PGEs on the whipping time of RDC. Uppercase letters indicate differences between concentrations, and lowercase letters indicate differences between PGEs.

In parallel to the stability of the emulsion, the whipping process also requires the ability to encapsulate the air churned in to form a firm foam, which determines the first impression of the consumer. This process consists of three stages, namely, rapid aeration, envelope bubble, and stabilization, with surface-mediated partial agglomeration and the adsorption of milk proteins considered to be key factors [20]. The variations in whipping time with the addition of the PGEs for the different RDCs are shown in Figure 4. The addition of the four PGEs significantly reduced the time required for aeration (*p* < 0.05), which was due to the emulsifier reducing the tension at the gas/liquid interface and contributed to the stabilization of the bubbles. However, there were differences in the trends of the emulsifier concentration and stirring time, and the stirring time of PMS did not decrease with an increase in its concentration (*p* > 0.05); this was because the action of the emulsifier was limited by the adsorption on the foaming surface [33]. However, the potential values also indicated no significant difference between the different concentrations of PMS. It is worth mentioning that the TML cream required the longest aeration time, which may be due to the synergistic effects between TML and proteins, as well as the high protein content at the fat globule interface leading to difficulties in forming a network structure to encapsulate air [33]. Sakamoto et al. showed that the heterogeneous nucleation of fats in oil/water emulsions is activated at the oil/water interface, where the template effect of the hydrophobic long saturated fatty acid chains of a PGE may play the main role in heterogeneous nucleation [34]. Similarly, there was less crystallization of TML as a heterophase core at the interface, and it was more difficult for the fat crystals to pierce the fat globules in order to interconnect.

### 3.6. Effect of PGEs on Overrun of RDC

**Figure 5:**
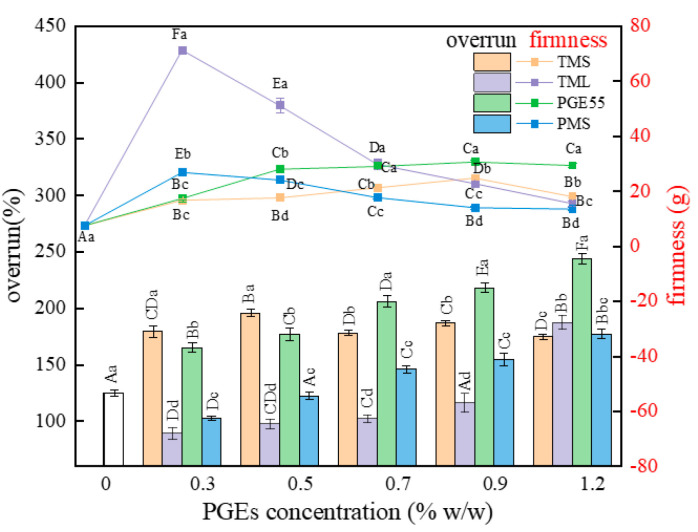
Effect of different PGEs on the overrun (the bar) and firmness (the line) of RDC. Uppercase letters indicate differences between concentrations, and lowercase letters indicate differences between PGEs.

The RDC churning process involves the emulsion encasing the churned-in air to form a stable solid foam, which essentially transforms from an O/W emulsion system to a foam structure stabilized by the agglomeration of proteins and fats [35,36]. The overrun of the RDCs with different PGEs is shown in Figure 5. At the end of the churning, the RDC without the PGEs appeared to be liquid in nature despite the formation of a large number of bubbles, while the samples with the PGEs formed a solid structure with some plasticity after a short period of churning. On the one hand, the addition of the PGEs increased the viscosity of the emulsion and hindered the migration of bubbles; on the other hand, it reduced the gas/liquid surface tension and increased the resistance of the foam to deformation [37].

The effect of the different PGEs on the overrun of the RDC showed considerable differences, and there was a certain negative correlation with the stability of the emulsion. As can be seen in Figure 5, the addition of 0.3% PGE55 and TMS significantly increased the overrun of the RDC to 165.14% and 179.66%, respectively, while TML and PMS only increased it to 89.50% and 102.52%, respectively. Although TML and TMS had the same degree of polymerization, the difference in fatty acids resulted in TML creams with a smaller particle size, a lower viscosity, and a lower ability to encapsulate foam. In addition, Fredrick et al. reported that the addition of TMS made it easier for triglycerides to form smaller crystals that could not pierce the oil/water interface and that contributed to the stability of the foam, giving it the highest overrun [4]. In addition, the overrun of TML and PMS increased with increases in their concentrations, which verifies the speculation that TML and PMS were weak in interfacial adsorption. The study conducted by Schmidts et al. also showed that the hydrophilic and lipophilic balance of emulsifiers affects the performance [38], while the HLB of TML and PMS was weak in lipophilicity due to short fatty acid chains or high polymerization, with HLB values of 8.5 and 11.3, respectively. Moreover, the adsorption intensity was increased with an increase in the concentration.

### 3.7. The Effect of PGEs on Firmness of RDC

The firmness of the whipped cream was measured using a texture instrument, and the results of the different PGEs are shown in Figure 5. The addition of the four PGEs significantly improved the firmness of the samples, with even a 0.3% concentration of the PGEs having a noticeable effect compared to the RDC without any PGE (*p* < 0.05). This may be due to the fact that, although PGEs have stronger emulsification and interfacial mobility, they reduce the interfacial strength when adsorbed at the oil/water interface, leading to an increase in crystal bridging [29]. The effect of the different concentrations and types of PGEs on firmness varied, with TML and PMS firmness decreasing and TMS and PGE55 firmness increasing with increases in their concentrations. The firmness of the TML and PMS creams was the highest at the 0.3% concentration, with 71.27 g and 26.83 g, respectively, while the hardness of the TMS and PGE55 creams was the highest at 0.9% and 1.2% concentrations, with 24.80 g and 30.67 g, respectively. On the one hand, this was associated with the overrun, where higher swelling rates contained more air bubbles and lower hardness. On the other hand, this verifies that different fatty acid compositions and polymerization degrees changed the role of the emulsifiers in the RDC emulsions and the aeration process through the hydrophilic and lipophilic balance of the PGE. The firmness of TML at low concentrations was of interest and provides ideas for the preparation of high firmness foams.

### 3.8. Effect of PGEs on Serum Loss of RDC

The amount of serum loss is an important indicator of the response to RDC plasticity and stability, and the effects of the different PGEs on serum loss are shown in Table 1. The addition of the PGEs significantly reduced the serum loss of the RDC compared to the control group (44%). This was because the addition of the PGEs reduced the size of the fat globules and increased the viscosity, which facilitated the formation of a fatty network structure to encapsulate the blisters and limit the pooling and efflux of the liquid phase [39]. The role played by the different types of PGEs varied, with TMS and TML creams not leaking at 0.9% and 0.7% concentrations, respectively, and with no leaks occurring at any concentration of PGE55. In contrast, the serum loss of the RDC was still as high as 7.9% at a PMS addition of 1.2%. This suggests that the PGEs with low degrees of polymerization helped to maintain the stability of the bubbles. Sakamoto et al. also reported the effects of fatty acid fractions and esterification degrees on PGEs and found that the hydrophobicity of PGFEs affects the crystallization behavior of heterogeneous nucleation at the oil/water interface [34]. Moreover, the serum loss of TML was lower than that of TMS at low concentrations, which might be due to the different fatty acids.

### 3.9. Effect of PGEs on the Partial Agglomeration Rates of Fat in RDC

Fat globules come into contact and collide with each other in a liquid due to their moving without restraint, agglomeration occurs between them, etc. Partial agglomeration depends on the size and surface properties of the fat globules [20,31]. The addition of the PGEs changed the surface properties of the fat globules, which contributed to the reduction in the partial agglomeration rate, and the results are shown in Table 2. The addition of the PGEs decreased the rate of fat aggregation in the RDCs, indicating that the PGEs competitively adsorbed into the reconstituted interfacial membrane after homogenization and played a positive role, which is consistent with the change in the liquid-phase protein concentration. Notably, the partial aggregation rate of the PMS emulsions was significantly higher than that of the TMS and PGE55 emulsions, indicating that the higher polymerization degree of the PGEs had less of an effect on partial aggregation. Similarly, the particle size of the PMS emulsions was larger, and the zeta potential did not change significantly, both of which indicate that PMS has a relatively weaker stabilizing effect at the interface because of its larger molecular weight and greater hydrophilicity. In addition, TML had limited ability to displace interfacial proteins, and the synergistic effect between the oil/water interface and the presence of proteins may contribute to enhanced interfacial stabilization; therefore, it had the lowest partial agglomeration rate.

### 3.10. Effect of PGEs on the Serum Protein Content of RDC

**Figure 6:**
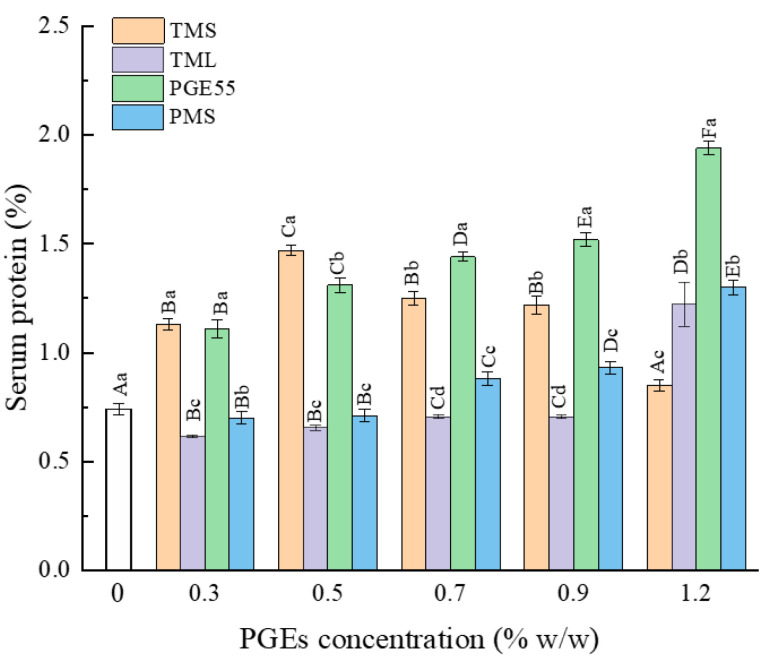
Effect of different PGEs on serum protein content. Uppercase letters indicate differences between concentrations, and lowercase letters indicate differences between PGEs.

The fat in an emulsion is dispersed in the liquid phase as microspheres and is stabilized by amphiphilic proteins adsorbed at the oil/water interface to form a viscoelastic surface layer [40]. The addition of surfactants creates a fluid adsorption layer that promotes droplet stabilization through the Gibbs–Marangoni effect. However, the presence of surfactants competes for adsorption at the oil/water interface, resulting in elevated protein levels in the liquid phase [41]. Thus, the level of proteins in the liquid phase can be used to determine the interfacial adsorption and the stability of the fat globules. The effects of the different PGEs on the concentration of proteins in the liquid phase are shown in Figure 6. Overall, the concentration of proteins in the liquid phase increased gradually with an increase in the PGE concentration. In the range of 0.3–1.2%, the addition of both TMS and PGE55 increased the serum protein concentration, while low concentrations of TML and PMS decreased the protein content, probably because TMS and PGE55 showed stronger lipophilicity and efficiently replaced interfacial proteins, even at low concentrations. In contrast, TML and PMS showed greater hydrophilicity and acted synergistically with the proteins at low concentrations, contributing to the adsorption of interfacial proteins; hence, a decrease in the liquid phase protein content occurred [42]. As the concentration increased, the competitive adsorption abilities of TML and PMS increased, displacing interfacial proteins through the orogenic displacement theory [29]; therefore, the serum protein content increased.

### 3.11. Effect of PGEs on the Microrheology Properties of RDC

**Figure 7:**
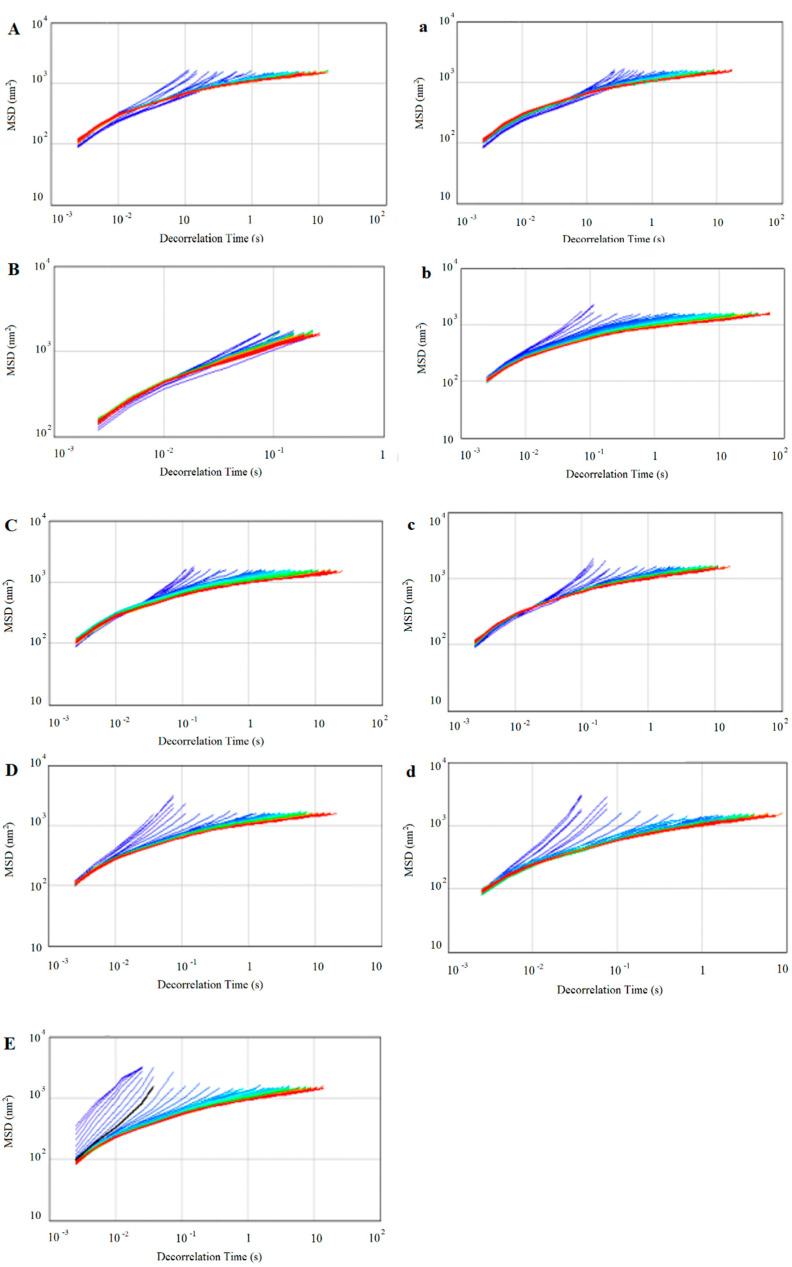
Microrheological curves of RDCs with different PGEs. (**A**–**D**) 0.7% concentration of TMS, TML, PGE55, and PMS; (**a**–**d**) 0.9% concentration of TMS, TML, PGE55, and PMS; (**E**) RDC without PGE.

Unlike shear rheology, microrheology analyzers calculate information on the velocity and displacement of particle motion by detecting the Brownian motion of particles under thermodynamic action, without the destruction of the emulsion structure [43]. The mean square displacement (MSD) represents the distance between the particle at a given moment and its initial position, and the MSD curves for the RDCs with the different PGEs added are shown in Figure 7. The MSD curves for all the RDCs showed a nonlinear relationship, which indicates that the RDC system was a viscous elastomer. The distribution of the MSD curves of the RDCs with the PGEs was denser than that of the MSD curves of the control. This could be attributed to the fact that the three-dimensional structure formed by the fat aggregates limited the movement of the particles; therefore, the particles had a smaller range of Brownian motion in the thin cream system [44], which manifested itself in increased viscosity and stability.

The elasticity index (EI) and macroscopic viscosity index (MVI) of the samples, indicating the elasticity and viscosity, respectively, can be calculated from the relationship curves of MSD and decorrelation time, as shown in Table 3. In addition to PMS, the PGEs caused the EI of the RDC to decrease and then increase, indicating that the addition of the emulsifiers made them less elastic. This probably resulted from the higher molecular weight of PMS and the thicker adsorption layer after competitive adsorption onto the surface of the fat globules [21]. The MVI reflects the viscosity of the system, meaning the time that it took for the particles in the system to reach the same position. The addition of any type of PGEs increased the MVI of the RDC, illustrating that the addition of the emulsifier limits the Brownian motion of the particles, a result that is consistent with the viscosity. The MVI of TML, TMS, and PGE55 increased with increases in their concentrations, while PMS decreased slightly at higher concentrations. This was presumably caused by the fact that the larger molecular weight of PMS allows it to adsorb less at the interface, and, at high levels, unadsorbed PMS then causes aggregation of fat globules and destabilizes the structure.

## 4. Conclusions

In summary, the different types of PGEs played different roles in the RDC, influenced by the structure. All PGEs improved the stability of the emulsions, and the increased MVI in the microscopic rheological analysis indicated that the addition of the PGEs enhanced the spatial potential resistance of the particles and limited the Brownian motion of the particles. Specific concentrations of the PGEs gave the RDC good aeration performance, but the different types of PGEs resulted in different performances due to different polymerization degrees and fatty acids. Concentrations of 0.7–0.9% of PGE55 and TMS gave RDC good churning performance and rapid expansion within 100 s (218.24% and 186.88%, respectively), making them a good choice to improve the quality of RDC. The high polymerization degree of PMS, however, had a poor effect on the emulsion stability and stirring characteristics at any concentration, which might be related to its complex molecular structure and poor adsorption ability at the interface. In contrast, the RDC with 0.9% TML, despite having the best stability, required a longer stirring time (318 s) and had a lower spillage (116.6%), and highly polymerized PMS performed poorly at any concentration, indicating that the high polymerization of the PGEs affected the aerated expansion of the RDC. Moreover, the low content of TML (0.3–0.5%) gave the cream a good hardness (71.27 g), which offers the possibility to develop highly plastic products.

## Figures and Tables

**Table 1:** The effect of the concentration of PGEs on serum loss.

Concentration (%)	TMS (%)	TML (%)	PGE55 (%)	PMS (%)
0.0	44.00 ± 0.03 ^Aa^	44.00 ± 0.03 ^Aa^	44.00 ± 0.03 ^Aa^	44.00 ± 0.03 ^Aa^
0.3	7.60 ± 0.06 ^Bb^	1.67 ± 0.59 ^Bc^	0.00	18.70 ± 0.37 ^Ca^
0.5	5.90 ± 0.08 ^Cb^	1.26 ± 0.53 ^Bc^	0.00	20.01 ± 0.39 ^Ca^
0.7	3.47 ± 0.05 ^Db^	0.00	0.00	27.32 ± 0.43 ^Ba^
0.9	0.00	0.00	0.00	18.68 ± 0.42 ^Ca^
1.2	0.00	0.00	0.00	7.90 ± 0.01 ^Da^

Data in the table are expressed as the mean ± SD (*n* = 3); uppercase letters indicate differences between concentrations, and lowercase letters indicate differences between PGEs.

**Table 2:** The effect of the concentrations of PGEs on partial agglomeration rates.

Concentration (%)	TMS (%)	TML (%)	PGE55 (%)	PMS (%)
0.0	8.95 ± 0.24 ^Aa^	8.95 ± 0.24 ^Aa^	8.95 ± 0.24 ^Aa^	8.95 ± 0.24 ^Aa^
0.7	4.14 ± 0.29 ^Bb^	3.73 ± 0.36 ^Bb^	3.67 ± 0.41 ^Bb^	7.93 ± 0.34 ^Ba^
0.9	4.67 ± 0.32 ^Bb^	2.65 ± 0.32 ^Cc^	3.03 ± 0.36 ^Bc^	7.34 ± 0.36 ^Ca^

Data in the table are expressed as mean ± SD (*n* = 3); uppercase letters indicate differences between concentrations, and lowercase letters indicate differences between PGEs.

**Table 3:** The effect of the concentration of PGEs on the EI and MVI.

	Concentration	TMS	TML	PGE55	PMS
EI (× 10^−3^)	0%	1.79 ± 0.03 ^Aa^	1.79 ± 0.03 ^Aa^	1.79 ± 0.03 ^Aa^	1.79 ± 0.03 ^Aa^
	0.7%	1.68 ± 0.03 ^Bb^	1.53 ± 0.04 ^Cc^	1.72 ± 0.01 ^Bb^	1.81 ± 0.010 ^Ba^
	0.9%	1.70 ± 0.08 ^Ab^	1.71 ± 0.01 ^Bb^	1.77 ± 0.02 ^Aab^	1.84 ± 0.01 ^Ba^
MVI (× 10^−3^)	0%	1.79 ± 0.03 ^Aa^	1.79 ± 0.03 ^Aa^	1.79 ± 0.03 ^Aa^	1.79 ± 0.03 ^Aa^
	0.7%	1.81 ± 0.01 ^Bc^	5.68 ± 0.10 ^Ba^	5.9 ± 0.17 ^Ba^	5.33 ± 0.14 ^Cb^
	0.9%	1.84 ± 0.01 ^Cd^	8.19 ± 0.18 ^Cb^	11.92 ± 0.30 ^Ca^	3.39 ± 0.17 ^Bc^

Data in the table are expressed as the mean ± SD (*n* = 3); uppercase letters indicate differences between concentrations, and lowercase letters indicate differences between PGEs.

## Data Availability

Data is contained within the article.
